# Role of senescent cells in the motile behavior of active, non-senescent cells in confluent populations

**DOI:** 10.1038/s41598-022-07865-2

**Published:** 2022-03-09

**Authors:** Thamara Liz Gabuardi, Hyun Gyu Lee, Kyoung J. Lee

**Affiliations:** Department of Physics, Korea University, Seoul, Korea

**Keywords:** Motility, Cellular motility, Computational biophysics

## Abstract

Characteristics of cell migration in a confluent population depend on the nature of cell-to-cell interactions as well as cell-intrinsic properties such as the directional persistence in crawling. In addition, biological tissues (or cell cultures) almost always carry anisotropies and they too can significantly affect cell motility. In the light of this viewpoint, the emergence of cellular senescences in a confluent population of active cells raises an interesting question. Cellular senescence is a process through which a cell enters a permanent growth-arrest state and generally exhibits a dramatic body expansion. Therefore, randomly emerging senescent cells transform an initially homogeneous cell population to a “binary mixture” of two distinct cell types. Here, using in vitro cultures of MDA-MB-231 cells we investigate how spatially localized cellular senescence affect the motility of active cells within a confluent population. Importantly, we estimate the intercellular surface energy of the interface between non-senescent and senescent MDA-MB-231 cells by combining the analysis on the motile behaviors of non-senescent cells encircling senescent cells and the result of extensive numerical simulations of a cellular Potts model. We find that the adhesion of normal cells to senescent cells is much weaker than that among normal cells and that the ‘arclength’ traveled by a normal cell along the boundary of a senescent cell, on average, is several times greater than the persistence length of normal cell in a densely packed homogeneous population. The directional persistent time of normal cell during its contact with a senescent cell also increases significantly. We speculate that the phenomenon could be a general feature associated with senescent cells as the enormous expansion of senescent cell’s membrane would inevitably decrease the density of cell adhesion molecules.

## Introduction

Tissue dynamics, or cell motility at the scale of individual cells, is important for various biological functions, being vital not only for morphogenesis^1–4^ but also for the homeostatic maintenance of various parts in living organisms. The immune response involves active migration of many different immune cells (e.g., neutrophils, macrophages, dendritic cells, and lymphocytes) to the wound sites, inflammation, or external intrusion^5–9^. Another good example is the regeneration and repair in the intestine, which are the most regenerative organ, replacing its epithelium lining every week or so. This process also involves numerous non-epithelial as well as epithelial cell types^10^. Yet another important example of cell motility, which is often collective, is the invasion of cancer^2,11,12^. All these biological phenomena share a common feature that they are *complex*, where one of its reasons being anisotropy. Most biological systems, in general, involve different types of cells having significant phenotypic variations, different levels of active motility, and different secretions^13^. Only a proper orchestration of all these factors would confer appropriate maintenance of relevant biological function.

Here we are interested in characterizing the active cell motility within a confluent monoclonal population of cells having (one specific kind of) localized anisotropies. The system of our current interest is simple: dense monolayer of MDA-MB-231 breast cancer cells in culture containing cellular senescence. Our recent experimental works showed that populations of monoclonal MDA-MB-231 cell lines in cell culture, in general, spontaneously develop a small subpopulation of cellular senescences, which can undertake some active roles in tissue-level structural transformation^14^. In 2D cell culture, MDA-MB-231 cells undergoing cellular senescence phenotypically distinguish themselves from non-senescent (normal) cells rather clearly with an enormously (sometimes over 100-fold) expanded cell area, thus, they are often referred to as “fried eggs”^14–18^. With the dramatic phenotypic transformation brought by cellular senescence, we presume that the expanded cell surface would have its cell adhesive property quite different from that of normal cells^16,19^.

Earlier, it was reported that embryonic cells of two different types that are initially dissociated and randomly mixed can even spontaneously sort to reestablish homogeneous binary phase separations, even when the involved cells do not actively crawl around^20^. Subsequently, the difference in the intercellular adhesive property of the two cell subpopulations, in connection with a Cellular Potts Model (CPM), was attributed to this sorting phenomenon^21^. In the simulation of a CPM, the final configuration of cell populations approaches to a state having the global minimum of the total energy associated with the system, of which intercellular surface energy (which determines the “stickiness” of interface) is one of the main factors affecting the tissue (or confluent cell population) dynamics. In fact, it was suggested that different intercellular surface energies for different interfaces in a binary mixture of cells that led to a complete cell sorting^20,22^. In other cases, different levels of surface energies could lead to different flocking behavior of homogeneous cell populations^19,23,24^.

In this paper, we carefully estimate the intercellular surface energy *E*_*sn*_ between normal and senescent MDA-MB-231 cells by combining the result of numerical simulations of a (previously validated) CPM and experimental analysis on the angular speeds *ω*s of normal cells that move along the boundary of a senescent cell. We find that *E*_*sn*_ (~ − 20) is much higher than the intercellular surface energy between normal cells (*E*_*nn*_ ~ − 65); that is, normal cells form a much weaker adhesion to senescent cells than normal cells themselves. Consequently, each encounter of a normal cell with a senescent cell body provides a small, curvy trajectory, during which the directional persistent time (and length) has increased significantly.

## Results

### Motility of normal cells in contact with a senescent cell in experiments

**Figure 1:**
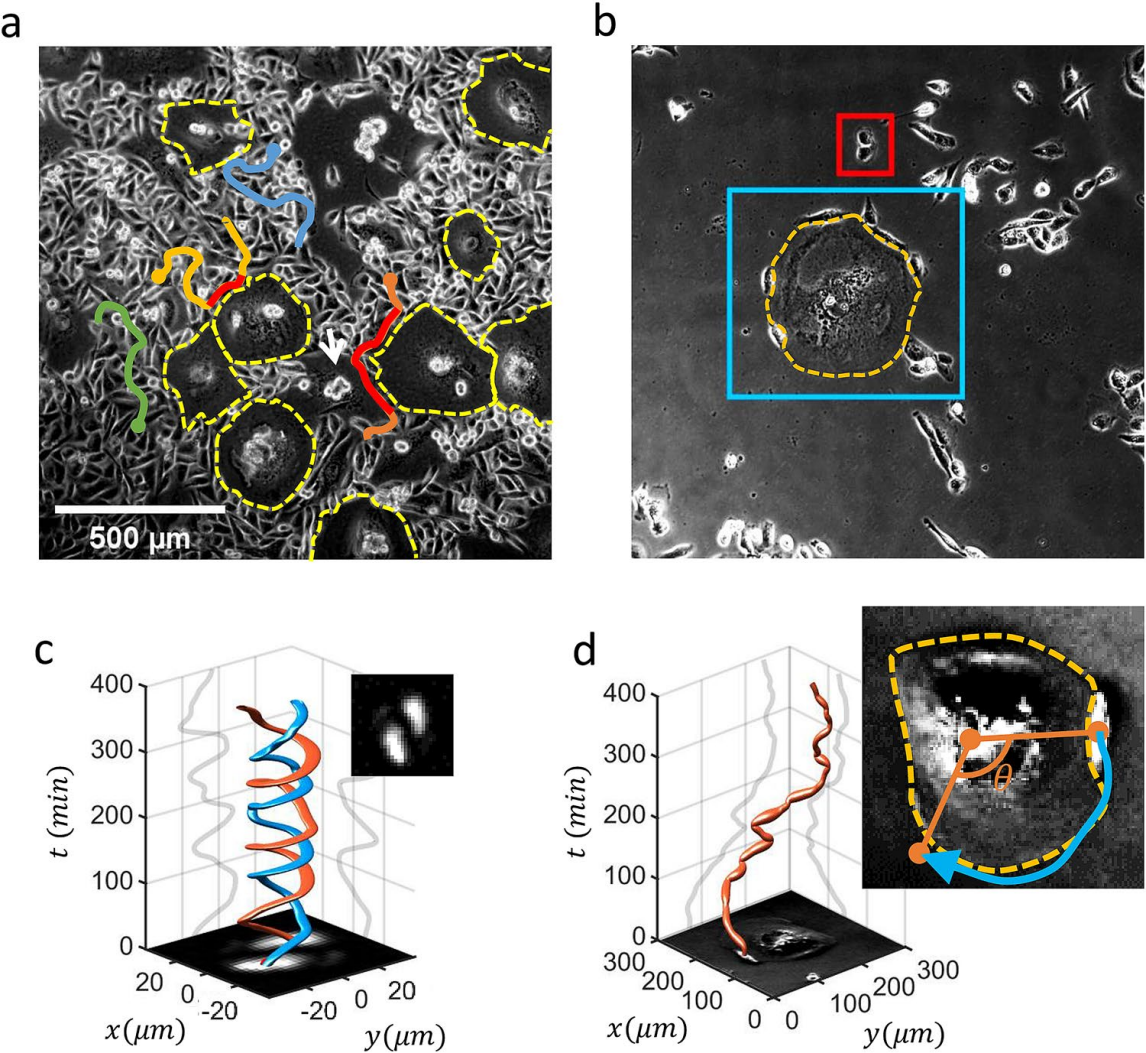
Binary (normal and senescent) mixtures of MDA-MB-231 cells and interesting pairwise cell motilities mediated by cell–cell adhesion. (**a**) A snapshot (phase-contrast) image of confluent monolayer MDA-MB-231 cell-culture (cell density = 526 cells/mm^2^) including several cells that have undergone cellular senescence [hugely enlarged areas that very much appear to be empty spaces, free of cells, bounded by yellow dashed lines]. Senescent cells emerge randomly in time as well as in space. Four exemplary moving trajectories of normal cells within the population are superimposed on the snapshot image, traced with a time interval of 1 min. (**b**) A snapshot image of MDA-MB-231 cell culture at a much lower density (28 cells/mm^2^), exhibiting ‘adhesive nature’ between adjacent cells. A pair of normal cells (bounded by a red box) and several normal cells travelling around a senescent cell (bounded by a blue box). (**c**) A pair of normal MDA-MB-231 cells showing active rotational movement. The centroids of the two cells are traced over 400 min. The thickness of the ‘colored tubes’ represents the instantaneous velocity of the rotating cell ($\overline{{v}}$ = 1.19 μm/min, ${\Delta v} =$ 0.83 μm/min). (**d**) One *normal* cell circling around an enlarged senescent cell (whose territory is marked by a dashed line). The snapshot images in (**c**) and (**d**) correspond to *t* = 0. The two gray lines in (**c**) and (**d**) are the projection of the cell traces on to the x–t and y–t plane. The cell trajectories in (**c**) and (**d**) were created using a MATLAB package “tubeplot” (by Anders Sandberg, http://www.aleph.se/Nada/Ray/Tubeplot/tubeplot.html, 2005).

For a given high-density MDA-MB-231 cell culture, senescent cells were observed to emerge spontaneously from the initial seeding in a random fashion both in space and time (Fig. 1a). Then, the cell bodies (including nuclei) of senescent cells expanded enormously on 2D culture substrate over a few days, and accordingly, their morphologies were often referred to as “fried eggs”: See the huge empty spaces, which are guided by yellow dashed lines in Fig. 1a. Few years ago we analyzed this phenomenon in detail and found a surprising role of senescent cells actively recruiting newly replicated non-senescent cells, which show mitotic cell-rounding, towards their body centers to form a small cell cluster (as marked by a white arrow in Fig. 1a)^14^. With the emergence of these senescent cells, we may view the monoclonal cell culture as a binary mixture of two different cell populations: that is, senescent vs. non-senescent, normal cell populations. Apart from their obvious phenotypic differences, we hypothesized that physical as well as biochemical interactions between senescent cells and normal cells would be different from those among the normal cells. Alternatively, the scatters of senescent cells could be viewed as large inhomogeneities embedded in the confluent population of normal cells. Then, a question naturally arises: How the presence of these large inhomogeneities would affect the motility of normal cells, which were known to be super-diffusive^19^. Four exemplary moving traces of normal cells are superimposed in the snapshot image Fig. 1a, and two (orange and yellow) of them clearly show a quite long period of contact time (marked by red color) with the senescent cells in their vicinity.

For the crawling normal cells, the territories occupied by senescent cell bodies were like an island to which normal cells could not invade (except for adjacent replicating cells showing mitotic cell-rounding^14^): See, for example, the orange-colored cell trace which “wraps” around the senescent cell territory demarcated by a red solid line in Fig. 1a. Interestingly, normal MDA-MB-231 cells appeared to have some preference to stay in contact with senescent cells, and this propensity was rather obvious in a low-density cell culture where cells prefer to form small clusters. Two different examples are highlighted by two (red and blue) bounding boxes in Fig. 1b: Two non-senescent normal cells in contact stay together forming a bounded pair within the small red boxed area, while several normal cells are migrating along the huge senescent cell’s boundary inside the blue boxed area. The bounded pair of normal MDA-MB-231 cells generically exhibit a quite robust steady rotation (with a period ~ 100 min) as in the representative example illustrated in Fig. 1c, and the underlying mechanism for the pair rotation was discussed in our (KL and HL) recent work^19^. Briefly, the phenomenon requires (1) an active crawling of the cells with a proper degree of directional persistence (strength and memory) and (2) a good degree of cell–cell adhesion, among other properties. Similarly, a normal cell migrating along the boundary (yellow dashed line) of a large senescent cell is given in Fig. 1d (see Supplementary Video 1); the cell’s moving velocity fluctuates during the time course of migration but is quite steady as we will discuss more shortly. The representative example given Fig. 1d clearly suggests that the normal MDA-MB-231 cells have a preference to stay in contact with senescent cells. Then, the main question is how much the preference of cell–cell adhesion has changed due to cellular senescence. With a careful analysis of many crawling trajectories of normal cells moving around senescent cells, which have a wide range of sizes, and those of computer simulations of a Cellular Potts Model, we provide an answer to this question.

**Figure 2:**
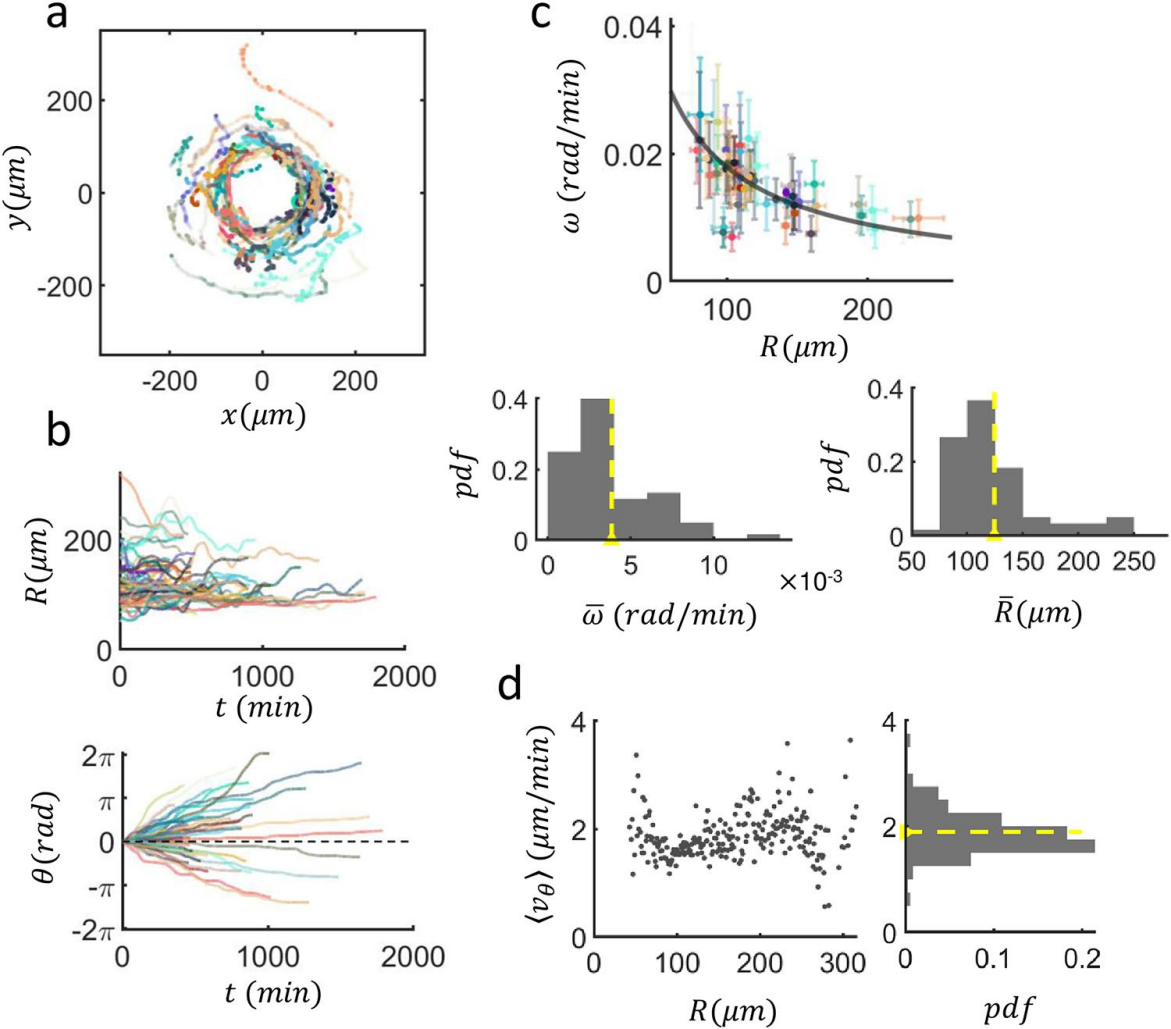
Analysis results showing heterogeneous motility of non-senescent cells and $\omega$’s functional dependence on *R*. (**a**) Trajectories of crawling MDA-MB-231 cells (*n* = 60 different culture samples) in the reference frames of host senescent cells. (**b**) Distances between the normal cells and their corresponding host senescent cells (top) and unwrapped angular positions $\theta$ of normal cells with respect to the centers of the host senescent cells as a function of time (bottom) for *n* = 60 different pairs. All occasional abrupt reverse-turns are flipped to match the initial chirality (bottom). (**c**) (TOP) Angular speed $\omega$ ($\Delta \theta$ over $\Delta t$ = 1 min) as a function of *R* for all *n* = 60 cells; colors match with trajectories in (**a**). Error bars represent the standard deviation, while the mean value for each cell is represented by a dot. The solid line is a fit for $A/R$ with $A = 1.79 \pm 0.10$ μm/min (over 95% confidence level) (BOTTOM) Histograms of temporally averaged angular speeds $\overline{\omega }$ (LEFT, mean value 3.9 × 10^–3 ^± 2.6 × 10^–3^ μm/rad) and the distances *R* (RIGHT, mean value 124.30 ± 40.46 μm) of all cells. The yellow dashed lines mark the mean of the time-averaged values. (**d**) Scatter plot of instantaneous tangential speed $\langle v_{\theta } \rangle$ against *R* (left) and $\langle v_{\theta } \rangle$’s histogram (right) (here, $\langle \cdots \rangle$ represents an average over all samples).

A total of 60 cases with a similar configuration (one or a few normal cells moving along the boundary of a senescent cell) was quantified, and the trajectories of normal cells were traced and plotted in Fig. 2a with the centroids of senescent cells positioned at the origin). On average, the motility of non-senescent cells was very steady with only a very few reverse turns observed. The contact between the normal cell and senescent cell forming a pair rotation lasted a minimum of 400 min, with some cases reaching 1800 min. We have decomposed the trajectories into (*R*, *θ*) as shown in Fig. 2b. Since the senescent cell body is not an ideal disk defined with a fixed radius, *R*(*t*), the distance between the centroid of the normal cell and that of the senescent cell, fluctuates significantly in time. Nevertheless, a small systematic increase in *R* over a day or so can be seen in Fig. 2b (top). The angular position *θ* of a normal cell encircling is also quite noisy but exhibits a clear systematic trend over time (see Fig. 2b, bottom frame). The local slope is nothing but the instantaneous angular speed *ω*: It also fluctuates a lot in time but has a characteristic value for different senescent cell bodies having a different *R* as illustrated in Fig. 2c (top) in which a total of 60 different samples are quantified. The time-averaged value of *ω* shows a clear *A*/*R* dependence with a fitting constant of $$ A = 1.79 \pm 0.10 $$ μm/min, which is nothing but tangential speed. The wide range of *R* covered in Fig. 2c simply reflects the diverse sizes of the senescent cells pairing with a non-senescent cell; as such we do not have any control over the range of *R*. Shown at the bottom of Fig. 2c are the histograms of $$\overline{\omega }$$ and $$\overline{R}$$ of $$n = 60 $$ different cases. Later, we run our model simulation based on these average values of $$\overline{\omega }$$ and $$\overline{R}$$. The fairly nice fit of $$\omega \sim \frac{1}{R} $$ (black solid line) in Fig. 2c suggests that the instantaneous tangential speed $$v_{\theta } ( = R\omega = A = 1.79$$ μm/min) of moving normal cell along the boundary of a senescent cell is rather independent of the properties (such as size or age) of the senescent cell. Indeed, the ensemble average of $$v_{\theta }$$ is measured to be 1.89 ± 0.53 μm/min which is off by 5.59% (see Fig. 2d). In other words, we would expect no significant curvature effect at play influencing the value of $$v_{\theta }$$, meaning that $$v_{\theta }$$ is very much an inherent motile property of non-senescent cells. Subsequently, we hypothesize that the large senescent cell bodies are like a wall that does not form a strong physical bonding but attracts non-senescent cells only very weakly. A cellular Potts model (see “Methods” for details) was used to recapitulate these experimental results and validate our hypothesis. Importantly, the ensemble average of $$\overline{\omega }$$ (and $$\overline{R}) $$ was used in the CPM simulations to estimate the interfacial energy associated with the interface between senescent cells and normal cells.

### CPM simulations of a normal cell moving along a senescent cell

**Figure 3:**
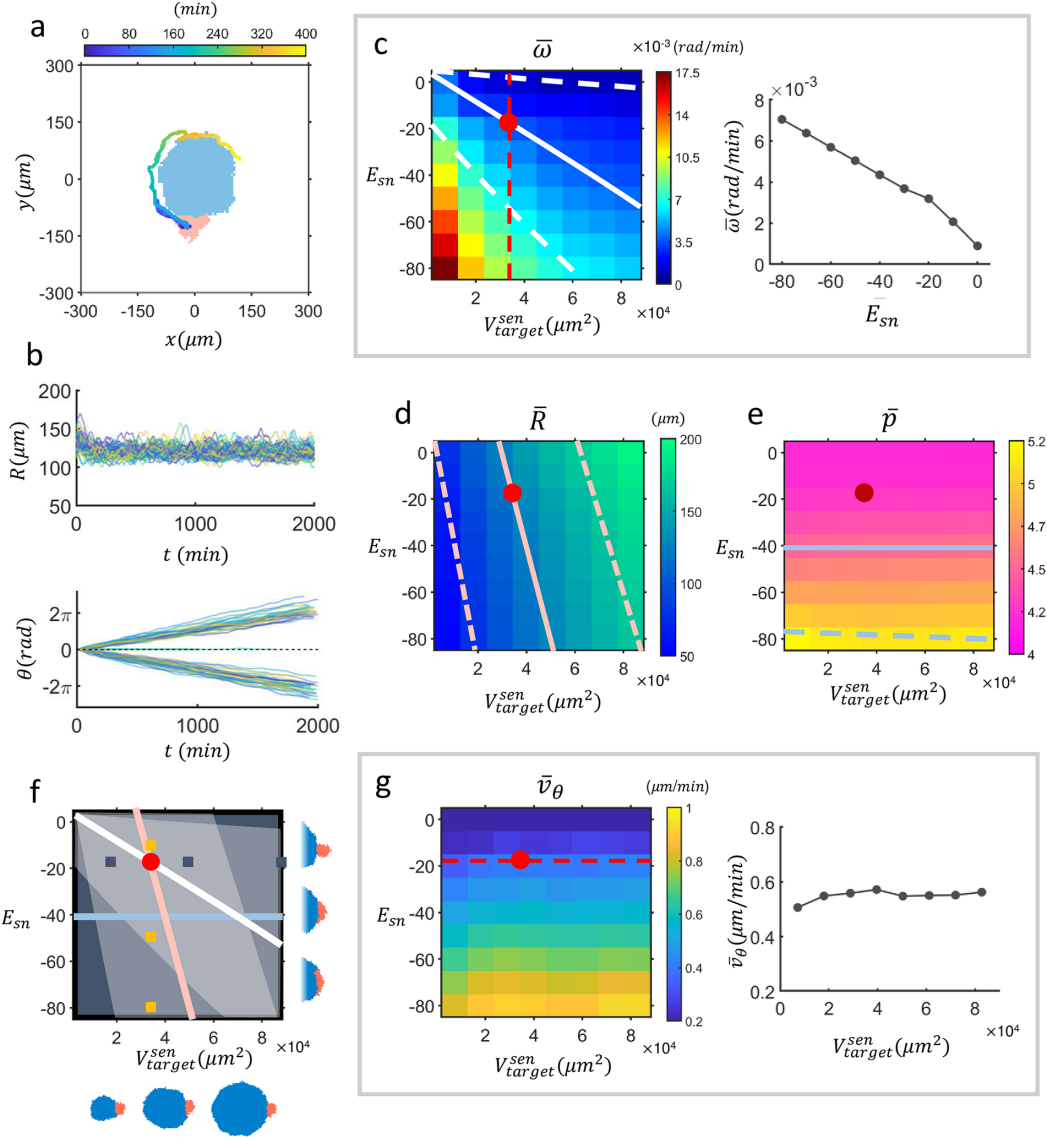
Identification of the adhesion energy *E*_*sn*_ for the interface between a normal cell and a senescent cell via cellular Potts model simulation. (**a**) An exemplary run showing a trajectory of a normal cell (pink), steadily circling around a senescent cell (cyan) cell (elapsed time is color-coded). (**b**) Distance *R* between normal cell and its hosting senescent cell (top) and unwrapped rotation angle $$\theta$$ of the normal cell (bottom) versus time (*n* = 60). (**c**) Phase diagram of (time as well as ensemble) average angular velocity given in a 2D plane of *E*_*sn*_ and the target volume of the hosting senescent cell $$V_{target}^{sen}$$. The white solid line is the level-curve of the experimentally measured value of $$\overline{\omega }$$ = 3.9 × 10^–3^ rad/min, and the dashed white lines mark the ranges of $$\Delta \overline{\omega }$$ = 2.6 × 10^–3^ rad/min. Shown in the right-hand side of the phase diagram is a scan of $$\overline{\omega }$$ along the vertical direction (red dashed line) set by $$V_{target}^{sen} = $$ 3.4 × 10^4^ μm^2^. (**d**) Phase diagram of $$\overline{R}$$: The pink solid (dashed) line marks the experimental value of $$\overline{R}$$ = 124.30 $$\mu m$$ ($$\Delta \overline{R}$$ = 40.46 μm). Shown in (**e**) is a phase diagram of CPM (normal cell shape) *p*-value; solid (dashed) blue line is the level curve of experimentally measured $$\overline{p} = 4.47$$ ($$\overline{p} = 4.47 + 0.6$$, mean + standard deviation). (**f**) Experimentally estimated 3 level curves of (**c**–**e**) overlaid on the phase diagram. Six representative cases of ($$E_{sn}$$, $$V_{target}^{sen}$$) are illustrated pictorially (cyan: senescent cell, pink: normal cell). Small yellow (navy) squares match with the side (bottom) snapshots in order. The red dot marks the set of parameter values (*E*_*sn*_ = − 20, $$V_{target }^{sen} = $$ 3.4 × 10^4^ μm^2^), with which CPM simulation results match the experimentally measured values of $$\overline{\omega }$$ and $$\overline{R}$$. We believe the experimental estimate of $$\overline{p} = 4.47$$ is less reliable, thus, not used for the estimation of *E*_*sn*_ (see text for detail). (**g**) Phase diagram of $$\overline{v}_{\theta }$$ and a scan of $$\overline{v}_{\theta }$$ along a horizontal direction set by $$E_{sn} = - 20$$ (red dashed line).

An appropriately set CPM could successively recreate the behavior of an active normal cell moving around a senescent cell as observed in experiments (see Fig. 3a and Supplementary Video 2). Recapitulation of the experimental results was carried out by assuming the same set of parameter values, which was validated in a previous study investigating MDA-MB-231 cell motility^19^ (see “Methods” for further detail). The given challenge in this work was to estimate a proper value of the interaction energy *E*_*sn*_ for the interface between two different types of cells: a large, non-motile (*S* = 0) senescent cell and a small, motile (*S* = 2.8), normal cell. The overall sizes of the two cells forming a pair were controlled by the parameters $V_{target}^{normal}$ and $V_{target}^{sen}$, where $V_{target}^{normal}$ was fixed to be 3600 μm^2^ and $V_{target}^{sen}$ was scanned as one of two control parameters. Figure 3b plots the distance *R*s (top frame) between the centroids of two cells forming a pair for *n* = 60 independent runs made with different initial conditions. Also shown in Fig. 3b (bottom frame) are the matching (unwrapped) rotation angles of the normal cell about the centroid of a senescent cell. Even for a fixed set of CPM parameter values, different runs would produce a small range of different rates of *θ* increase since there were stochastic events of reverse turns which temporally slowed down the overall rotation (see Fig. 3a and Supplementary Video 2). For the variable *θ* plotted in Fig. 3a, all such reverse turns were “unwrapped” so that the overall rate of *θ* (i.e., $\overline{\omega }$) increase could be visually more evident.

A phase diagram of $\overline{\omega }$ is drawn with two control parameters: $V_{target}^{sen}$, the targeted volume of the host senescent cell, and the interfacial energy *E*_*sn*_, controlling the level of adhesiveness at the interface of two cells forming a pair (see Fig. 3c). The solid (dashed) white line(s) in Fig. 3c represent the level curve(s) of the experimentally obtained $\overline{\omega }$ $( { \overline{\omega } \pm \Delta \overline{\omega }} )$. Likewise, we have constructed a phase diagram of $\overline{R}$ using the same set of control parameters as shown in Fig. 3d. For an obvious reason, the average distance ($\overline{R}$) between the two (senescent vs. non-senescent) cells forming an attached pair has a strong (weak) dependence on $V_{target}^{sen}$ ($E_{sn}$): In fact, *R* is mainly determined by the size of the senescent cell. The solid (dashed) pink line(s) in Fig. 3d represent the level curve (s) of the experimentally obtained $\overline{R}$ $( \overline{R} \pm \Delta \overline{R})$.

Another important feature of the cell pair shown in Fig. 3a is the shape of the normal cell crawling along the boundary of a senescent cell. Shown in Fig. 3e is a phase diagram of *p*-value (= $\frac{perimeter}{{\sqrt {V^{normal} } }}$), a dimensionless measure of cell shape which has been considered as an important characteristic of cell crawling behavior. On top of it, we have placed a level curve of (ensemble and time averaged) experimental *p*-value (4.47 ± 0.65, solid blue line), which is quite close to that (4.28) of the CPM parameter set of the “red dot” ($V_{target}^{sen} = 3.4 \times 10^{4}$, $E_{sn} = - 20$). But we should point out that the perimeter length, measured with experimental image data, can vary significantly depending on the way images are processed; obviously, a low-pass filtering will reduce the perimeter length (while not affecting the area much), subsequently leading to a smaller *p*-value. For the specified *p*-value (of the level curve), we have used only unprocessed raw images without any low-pass filtering. After all, $\overline{\omega }$ and $\overline{R}$ are considered as a more reliable benchmark than *p*-value.

Shown in Fig. 3f are two-level curves (one for $\overline{R}$ and the other for $\overline{\omega }$) intersecting at the position marked by a red dot. So, we have identified a particular value of interfacial energy $E_{sn} ( = - 20)$ which is experimentally most relevant for the inhomogeneous pair of MDA-MB-231 cells. Also illustrated alongside the level curves are six different configurations of the cell pairs, each of which corresponds to a square-marked position on the phase diagram. According to Fig. 3d, it is clear that $\overline{R}$ is almost linearly proportional to $V_{target}^{sen}$ but their proportionality changes as a function of *E*_*sn*_; also, we see that the curvature (or $V_{target}^{sen}$) of the boundary of the senescent cell barely affects the shape of the adherent normal cell, whereas *E*_*sn*_ significantly does so. The graph on the right-hand side of Fig. 3c shows that the value of $E_{sn}$ also affects $\overline{\omega }$ greatly (when the value of $V_{target}^{sen}$ is fixed). Finally, Fig. 3g shows a phase diagram of (time-averaged) instantaneous tangential speed $\overline{v}_{\theta }$, and one thing stands out clearly: $\overline{v}_{\theta }$ barely changes as a function of $V_{target}^{sen}$, which is consistent with the experimental result. In fact, it is *E*_*sn*_ that affects not only the shape of the moving non-senescent cell but also its tangential speed: The more the cell is elongated with a stronger adhesion the faster it migrates.

### Actively moving normal cells in the vicinity of a senescent cell in confluent population

**Figure 4:**
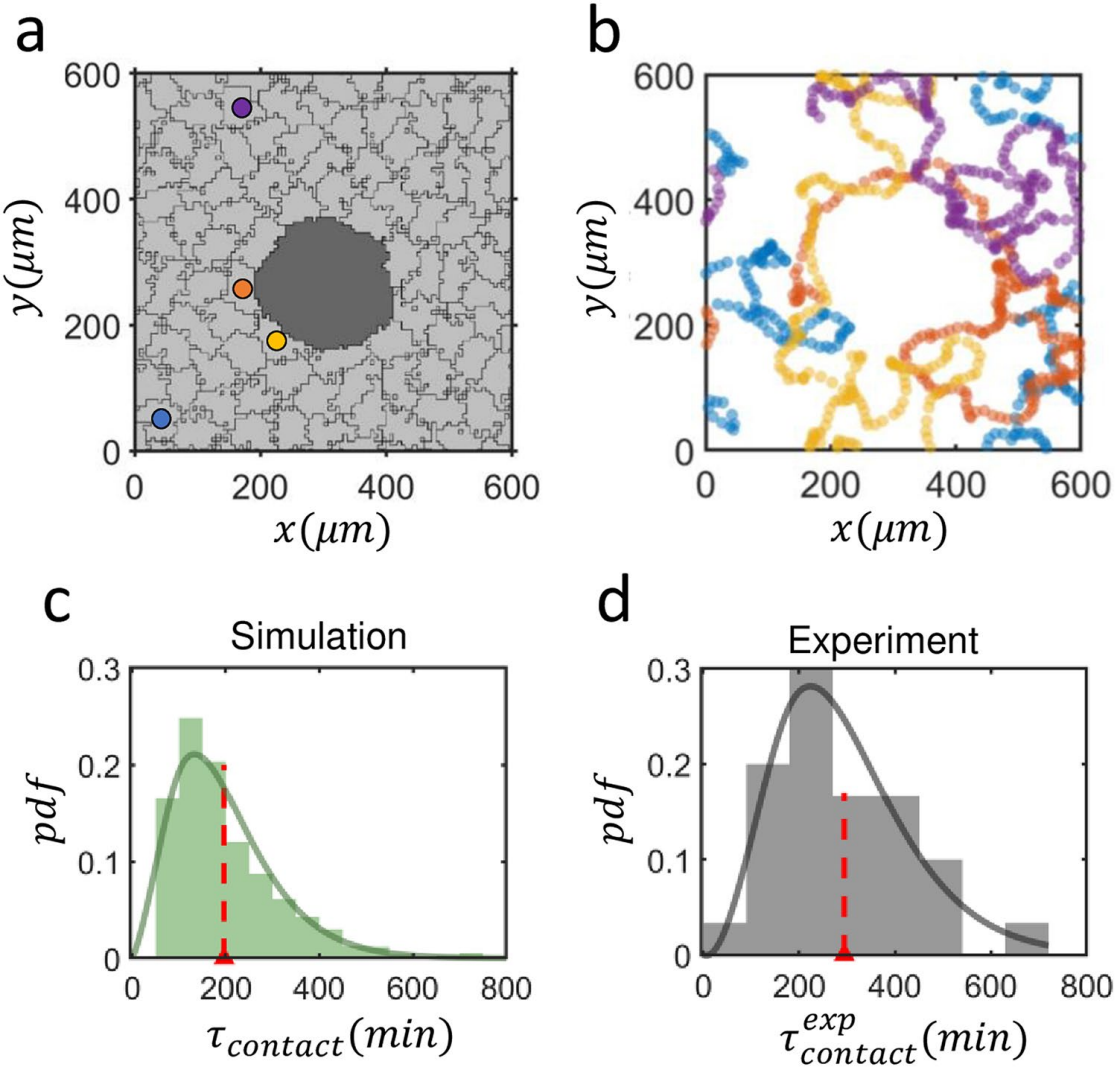
Contact-time statistics of the normal cells to senescent cells within confluent population. (**a**) A snapshot image of a CPM simulation: senescent cell (dark gray) and normal cells (light gray). (**b**) Color-matched trajectories of 4 randomly picked cells marked in (**a**) interacting with the senescent cell. A total of 91 non-senescent normal cells and 1 senescent cell filled space without any voids under a periodic boundary condition. The simulations are conducted with *E* = − 20 and $$V_{target} = $$ 3.4 × 10^4^ μm^2^ for 10^4^ MCS. (**c**) Histogram of a population/ensemble-averaged contact duration $$\tau_{contact}$$ (196 ± 122 min, mean ± sd) between normal and senescent cell pairs over 30 different runs with different initial conditions. The solid line draws fitted gamma function with coefficients $$\alpha = 3.17, \;\beta = 62.00$$. (**d**) Histogram of the contact duration $$\tau_{contact}^{exp}$$ (*n* = 30, 294 ± 140 min, mean ± sd) measured in experiments. Fitted gamma function with $$\alpha = 4.22, \;\beta = 69.75$$ is shown as dark gray line. The red dashed lines in (**c**) and (**d**) indicate the mean values.

The first application of CPM in biology was to explain the phenomenon of “phase separation” between two different kinds of cell populations that had different intercellular surface energies for different types of interfaces. Here, we have a very similar issue: How might the motility of non-senescent cells be modified when there are large senescent cell bodies scattered in their confluent population. Having figured out the experimentally relevant set of parameter values ($E_{sn} = - 20$, $V_{target} = 3.4 \times 10^{4}$ μm^2^), we are now ready to run a realistic simulation as illustrated in Fig. 4a (also see Supplementary Video 3). A large senescent cell body, which can move only passively, is placed in the middle and barely moves. Four exemplary moving traces of the color-marked normal cells in Fig. 4a are given in Fig. 4b. All four marked cells in confluency have come across with the senescent cell in the middle and stayed in contact with it for some duration until they departed for elsewhere. The residence time upon each random visit to the senescent cell varied significantly from one to the other cases and its probability distribution fits well to a gamma distribution $(\alpha = 3.17,\; \beta = 62.00)$ as illustrated in Fig. 4c. The mean residence time was measured to be 196 min. For the same set of parameter values, but for the case of a single normal cell encircling a senescent cell, its residence time is almost infinite (not shown). And the significant reduction of the residence time in a confluent population is, of course, a consequence of normal-to-normal cell interaction which shows a much stronger adhesion with $E_{nn} \sim - 65$^19^. As in the CPM simulation results of Fig. 4c, we have measured the residence time $\tau_{contact}^{exp}$ of normal cells (*n* = 30) that are in contact with a senescent cell in experiments, and its distribution is given in Fig. 4d. Like its CPM counterpart of Fig. 4c, the pdf fits a gamma function reasonably well $(\alpha = 4.22,\; \beta = 69.75)$ with a mean of 294 min, which is about 50% larger than that of its CPM counterpart; and we speculate that senescence-associated secretory phenotypes (SASPs) could be attributed to this increment as discussed in^14^.

**Figure 5:**
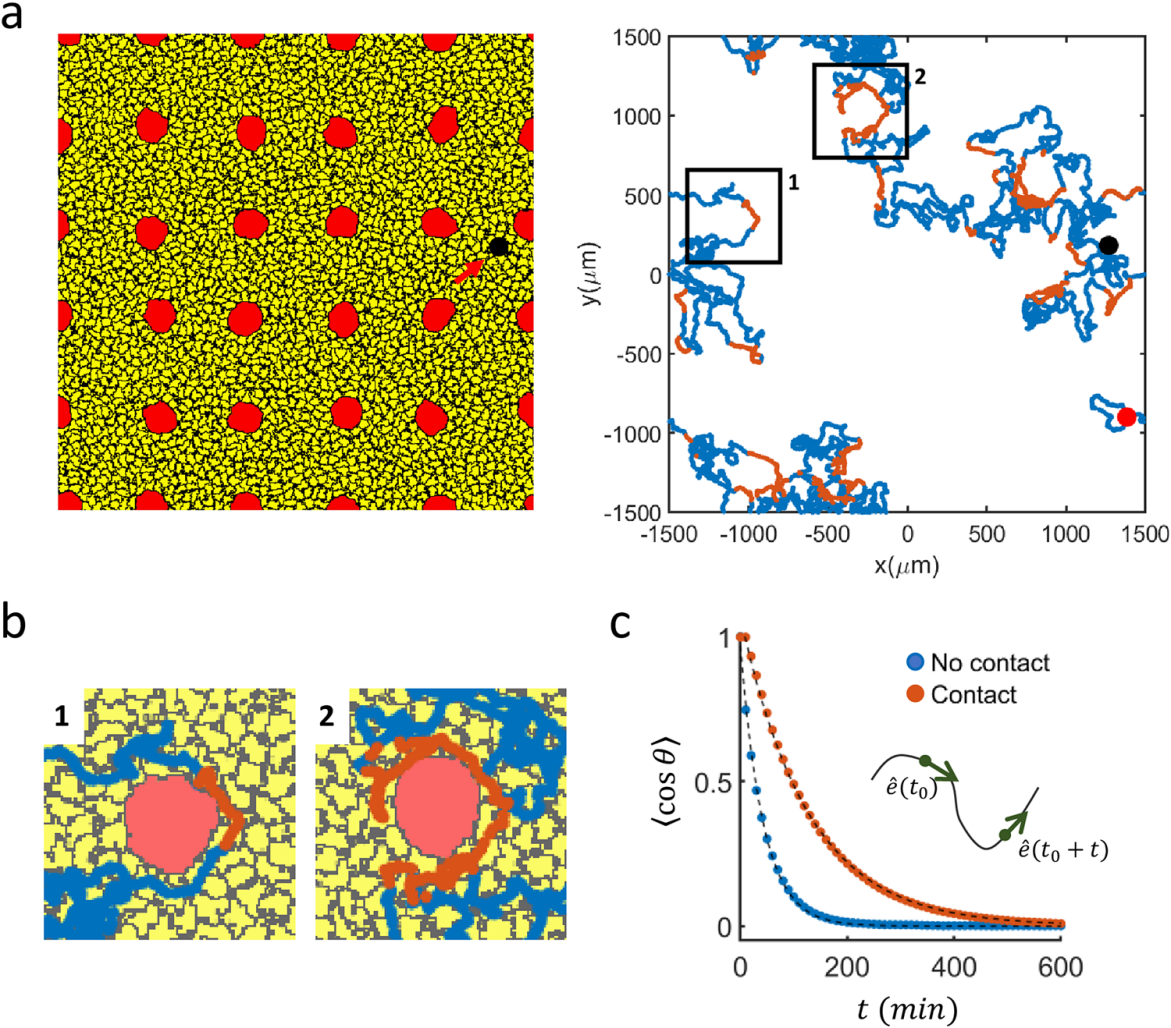
Influence of senescent cells on the moving trajectories of normal cells in confluency. (**a**) A snapshot image of a CPM simulation: (non-active) senescent cells (red) and (active) normal cells (yellow). One normal cell (randomly selected; marked by block dot) is traced in the right-hand side (red line: during a contact with a senescent cell; blue: during no contact with senescent cells). (**b**) Two blown-up insets of (**a**). Senescent cells move only a little passively. (**c**) Average dot product of two directional vectors separated by time *t* (see the schematic illustration). $\tau_{no\_contact} ( = 43.42$ min) and $\tau_{contact} ( = 123.50$ min) are a decay time constant of the corresponding exponential fit. The image in (**a**) was generated by Morpheus 2.2 (https://morpheus.gitlab.io).

We have also made CPM simulations mimicking the confluent binary mixture of cells in a larger domain to see how the motility of actively crawling normal cells would be altered in the presence of many large senescent cells (see Fig. 5a, left frame). As in the cases of heterogeneous cell pairs of Fig. 2a, the normal cells have a clear tendency to encircle the senescent cell on each encounter (see Fig. 5a, right frame): The blown-up insets shown in Fig. 5b are two exemplary cases. Clearly, the directional persistence of the cells moving along the perimeters of senescent cells are enhanced significantly as shown in Fig. 5c [persistence times: $\tau_{no\_contact} = 43.42$ (min) $\tau_{contact} = 123.50$ (min)]. In the meanwhile, instantaneous speed has no difference for the two different cases, and this seems to be a reminiscence of the fact that $v_{\theta }$ does not depend on *R* for the cases of heterogeneous pair of cells. Consequently, each encounter of a normal cell with a senescent cell body adds a small (curved) trajectory; yet it does not cause a significant ‘packing density’ fluctuation in time^25^ (along the moving trajectory) as $\tau_{contact}$ is only about three times that of $\tau_{no\_contact}$. As expected, both $\tau_{contact}$ and $l_{contact}$ (contact length along the boundary of senescent cell) increase monotonically as a function of $E_{sn}$ (and $V_{target}^{sen}$). That is, the less sticky the interface is (or the larger the senescent cell body gets), the longer $\tau_{contact}$ and $l_{contact}$ become (see Supplementary Fig. S1).

## Summary and discussion

To understand the effects of cellular senescence on the motile behavior of MDA-MB-231 cells in confluency, we followed a bottom-up approach where heterogeneous systems with a much simpler configuration, namely, a cell pair formed by a normal cell and a senescent cell, were compared to the previously studied homogenous counterpart, that is, a cell pair formed by two normal cells^19^. After carefully analyzing the trajectories of two different types of cells during many free encounters in the experiment, a CPM was set up to recapitulate the results of experimental observations. The same CPM simulation was repeated for many different sets of parameter values, and a suitable value for the interfacial surface energy relevant for the interface between the two different types of cells was estimated. A similar strategy was applied to confluent populations having inhomogeneities (i.e., senescent cells), with special attention given to the residence time (i.e., contact time duration) of the normal cell, during which it stayed in contact with the hosting senescent cell.

Unlike the pairwise rotation of normal cell pairs, the movement of the normal cell around a hosting senescent cell was non-mutual. Moreover, the characteristics (size, shape) of the involved senescent cells varied significantly from one to another due to the inherent heterogeneity of the cultured sample. Importantly, our analysis showed a strong 1/*R* functional dependence for the normal cell’s angular speed $\omega$, while its tangential speed along the senescent cell boundary $v_{\theta }$ remained constant (i.e., invariant over a significant curvature variation).

Based on the average values of $\omega$ and R obtained in experiments, the adhesion energy *E*_*sn*_ between senescent-non-senescent cell pair interaction was estimated to be much higher (*E*_*sn *_= − 20) than that (*E*_*nn *_= − 65) of equivalent non-senescent homogeneous pair interaction reported in our recent study^26^. Then, the significantly large *E*_*sn*_ (compared to* E*_*nn*_) should be responsible for the nearly ballistic movements of non-senescent cells that are in contact with senescent cells. After all, for the normal MDA-MB-231 cells the boundaries of hugely expanded senescent cells merely acted like an impenetrable wall which was far less sticky than peer normal cells in the population. Although there are previous studies collectively suggesting a role for aberrant adhesion protein (e.g., integrin) signaling in senescence, but how cellular senescence modifies cell–matrix adhesion is poorly understood^27^. In the future, it will be important to identify the biochemical mechanism underlying the reduced interfacial surface energy caused by cellular senescence.

Within a confluent population on a 2D substrate, non-senescent MDA-MB-231 cells exhibit a “superdiffusive” motility, where the directional superdiffusivity is even stronger than that of freely crawling cells^26^. Yet, the neighbor-enhanced persistence length was measured to be only 72 μm, which was about 1.2 times a cell diameter (cell diameter is assumed to be 60 μm), and the moving trajectory of a normal MDA-MB-231 cell could be viewed as a sequence of uncaging (or position swapping) events or position swapping of two adjacent cells. For the actively crawling normal cells, moving along the less sticky boundary of senescent cells would be energetically more favorable than joining into a densely-packed, stickier population: Therefore, the arclength along the senescent cell boundary covered over residence times (see Fig. 2a) was naturally far greater than the diameter of a normal cell. Then, the detachment of a normal cell from the boundary of the senescent cell could be incurred by a stochastic position swapping event with neighboring normal cell(s) as discussed in ref^19^.

## Conclusion

With the help of CPM simulations we found that senescent MDA-MB-231 cells, which emerged spontaneously and randomly in space and time in cell culture, act like large physical obstacles providing only a low level of stickiness for actively crawling normal MDA-MB-231 cells in confluent populations. Consequently, each encounter of a normal cell with a senescent cell body adds a small but significant piece of circular trajectory on top of already super-diffusive moving trajectories of normal cells in confluency. Although there are several different versions of CPMs incorporating different mechanisms, for example, for the directionally persistent motility, the CPM model that we have employed in this investigation was validated earlier in a previous study modelling the super-diffusive nature of MDA-MB-231 cells in confluency^19^. This CPM includes neither detailed biochemistry orchestrating the cytosolic actin dynamics nor the biochemical interactions mediating the interfacial adhesiveness; therefore, it is quite surprising and fascinating that this simple model could recapitulate the observed phenomena in a very quantitative manner. Lastly, we should point out that senescent cells, in general, are known to excrete various SASPs^16,17,28^ which can act as a chemo-attractant (or -repellant); a property that was attributed to the formation of cell clusters at the core of each senescent cell body in our previous study. In this work, we did not incorporate the potential chemotactic force that could have facilitated the adhesive property of the senescent cell boundary; and we speculate that it could have been an additional factor that made the average $\tau_{contact}^{exp}$ about 50% larger than the average $\tau_{contact}$ of CPM simulation. Finally, we should comment that although the 2D phenomenon that we discuss in this paper is conceptually important, most biological tissues and organs are three-dimensional. As such, we have a plan to investigate the same issue in more physiological 3D environments.

## Methods

### Cell culture

High-density culture of MDA-MB-231 cells (~ 70% confluency) growing in DMEM culture medium (10% FBS) on a 3.5 mm petri dish was trypsinized with Trypsin–EDTA solution 10 × (Cat. No. 59418C, Sigma-Aldrich) diluted to 5 × with DPBS (Cat. No. D8537, Sigma-Aldrich) for 3 min after removing the DMEM medium. The solution was then gently stirred, centrifugated, and the supernatant discarded. The remaining cells were washed with DPBS solution and centrifugated again; this washing procedure was carried out twice before the addition of 1 ml of DMEM solution to the final remaining cells. Following cell counting, two different samples that either have a medium initial confluence (60%) or low initial confluence (20%$$)$$ were prepared. All samples were stored in a laboratory incubator at 37 °C and 5% CO_2_ perfusion (for about one day for low-density samples and 4–5 days for high-density samples) until they were transferred to the microscope incubator chamber for observation.

MDA-MB-231 cells were purchased from Korean Cell Line Bank (KCLB No 30026).

### Live-cell imaging

A small, thin cylindrical, temperature-controlled chamber incubator was designed and lab-built for live-cell imaging over long periods. The inside of the chamber was kept at a constant temperature of 36 ± 0.1 °C with a heating coil controlled by a temperature controller (SDM9000, Sanup, Korea) equipped with a PT100 temperature probe. Two ITO-coated optical windows of the incubator along the imaging axis were also electrically heated to avoid condensation. A mixed gas of CO_2_ (5%) and air (95%) was continuously perfused to the incubator. The incubator was positioned and fixed on the x–y stage of an inverted microscope (Olympus IX710).

A sample petri-dish containing the designated samples of MDA-MB-231 cells in a proper medium (89% RPMI serum, 10% Fetal Bovine serum, and 1% Penicillin–Streptomycin) was then loaded into the chamber. Time-lapse phase-contrast images were acquired with time intervals of 1 min using ProgRes MFcool CCD Camera (Jenoptik, Germany) with 4 × (NA 0.13) phase-contrast objective lenses and Micromanager software. The camera has a sensor resolution of up to 1360 × 1020 pixels with a measurement precision of 2 µm. A mechanical shutter was used to block the light in between image acquisitions.

### Imaging processing

The crawling MDA-MB-231 cells in our experiments show a significant activity of pseudopods, whose existence can make them look more elongated and ramified than those of CPM simulations. Since these pseudopod activities seem irrelevant to the normal cell–senescent cell interface formation, (which is relevant to *E*_*sn*_ for the case of CPM), they are excluded in the cell-shape analysis. On the other hand, with the gray-scale phase-contrast images and the separation process relying on a (gray scale) threshold alone, it is not possible to clearly distinguish pseudopods from cell bodies as the boundaries between them are sometimes unclear. On top of the innate cellular inhomogeneity of MDA-MB-231 cells, this could be a significant factor contributing to the uncertainty in the measured *p*-value. Perhaps more importantly, the perimeter length can vary significantly depend on the way image was taken and processed. In this sense, $\omega$ and *R* are considered as a better benchmark than *p*-value, when they were compared with those of CPM simulations.

### The cellular potts model simulation

Cellular Potts Model (CPM) is a lattice-based model that represents a given biological cell as a group of connected lattice sites. Each cell is allowed to interact with the cell surroundings along its boundary (e.g., other cells, physical substrates and barriers, and medium). The lattice domain evolutions (e.g., cell–cell interaction) are governed by the system’s free energy. CPM evolves in time and space based on a Monte Carlo method, in which (1) one of the lattices is picked randomly; (2) subsequently, we select a ‘neighbor site’ randomly among all ‘surrounding sites’ of the chosen lattice site; (3) the probability $$P_{accept} $$ of the initially chosen site accepting the selected neighbor site’s cell-type is based on Metropolis probability [$$P_{accept} = 1$$ (if $$\Delta H < 0); \;e^{ - \Delta H/T} \;(if\;\Delta H > 0)],$$ where $$\Delta H $$ and $$T $$ represent the change in the total Hamiltonian and the level of cell membrane fluctuations^29^, respectively. The system would evolve towards the state of minimum energy. The $$\Delta H$$ includes the following three components:

$$ \Delta H = \Delta H_{constraints} + \Delta H_{adhesion} + \Delta H_{persistence} $$

$$ H_{constraints} = \lambda_{area} \mathop \sum \limits_{i} (A_{target} - A_{i} )^{2} + \lambda_{perimeter} \mathop \sum \limits_{i} (P_{target} - P_{i} )^{2} $$

$$ H_{adhesion } = \mathop \sum \limits_{interfacei,j} E_{{\sigma_{i} ,\sigma_{j} }} ( {1 - \delta_{ij} } ) $$

$$ \Delta H_{persistence} = - S \mathop \sum \limits_{i} \frac{{\Delta {\varvec{x}}_{{\varvec{i}}} \cdot {\varvec{p}}_{{\varvec{i}}} }}{{\sqrt {{\varvec{p}}_{{\varvec{i}}} \cdot {\varvec{p}}_{{\varvec{i}}} } }} $$

$$ \Delta {\varvec{p}}_{{\varvec{i}}} \user2{ } = \user2{ }\Delta {\varvec{r}}_{{\varvec{i}}} \user2{ } - \user2{ }\frac{{{\varvec{p}}_{{\varvec{i}}} }}{\tau } $$

where $A_{i}$ and $P_{i}$ are the current area and perimeter of the cell indexed by $i$, and $E_{\tau i, \tau j}$ is the interfacial (adhesion) energy. $\lambda_{area} ,\; \lambda_{perimeter} , \;A_{target} , \;P_{target} ,$ and $S$ are fixed parameters. $\Delta H_{adhesion}$ models the change in the energy conferring the persistence of moving cells. For the chosen set of parameter values that we used for Figs. 4 and 5, the size of $\Delta H_{constraints}^{P} = \lambda_{perimeter} \Delta \mathop \sum \nolimits_{i} (P_{target} - P_{i} )^{2}$ [$\Delta H_{constraints}^{A} = \lambda_{area} \Delta \mathop \sum \nolimits_{i} (A_{target} - A_{i} )^{2} ]$ is most (least) significant and $\Delta H_{persistence}$ comes second as for updating the physical state of each cell (see Supplementary Fig. S2 for further details). $p_{i}$ is the polarity vector of *i*’th cell, and $\Delta x_{i}$ the hypothetical displacement of the cell’s centroid if the neighbor’s lattice site is copied to the target lattice site. At each Monte Carlo step, $p_{i}$ evolves with the addition of $\Delta r_{i}$, which is the spatial displacement of the centroid at the current time, and the factor reflecting directional memory loss $- p/\tau$, where $\tau$ represents the time constant for the memory loss. If $\tau \to \infty$, $p_{i} ( t ) = p_{i} ( {t - 1} ) + \Delta r_{i}$, which means the current polarity vector depends on the entire history of the passage taken by the cell. If $\tau \to 1$ (i.e., the minimum time step), $p_{i + 1} ( t ) = \Delta r_{i}$.

All (non-senescent) model cells have directional persistence in their motility and some degree of “stickiness” with neighboring cells; so, in a confluent population, they can be viewed as super-diffusive particles interacting with one another via interfacial interaction^26^. On the other hand, senescent cells are deprived of self-persistent motion (i.e., the parameter associated with self-propulsion is zero), since the cell bodies of senescent MDA-MB-231 cells are in general very huge and they barely move in comparison with non-senescent tumor cells^14^. In this investigation, we have varied only two parameters in efforts to investigate the effect of the senescent cells' overall size (or the curvature of cell boundary in 2D space) and adhesiveness between the senescent and non-senescent cells: namely, senescent cell’s target volume $$V_{target}^{sen} $$ [and also $$P_{target}^{sen}$$ in proportion to $$( {V_{target}^{sen} } )^{1/2}$$] and adhesion energy $$E_{sen - cell} $$ between senescent and non-senescent cell boundaries. All other parameter values are fixed as following: $$\tau = 4$$, $$\lambda_{area} = 1$$, $$\lambda_{surface} = 1$$, $$A_{target} = 100$$, $$P_{cell} = P_{senescent} = 0.9$$, $$E_{cell{-}cell} = - 65, E_{medium {-}medium} = 0$$, $$E_{cell{-}medium} = 0$$, and the parameter $$T $$ was set to 10. Here, the subscript (or superscript) ‘$$sen$$’ and ‘$$cell$$’ refer to the senescent and normal cells, respectively. In the simulation, 1 MCS corresponds to updating sites the number of times equal to the number of total sites in the system and it was estimated to be 2 min in earlier work of ours^19^. All simulations were done inside a 600 × 600 square lattice space with periodic boundary conditions for over 10^4^ Monte Carlo steps (MCS) using a CPM simulation platform Morpheus^30^.

## Supplementary Information

Supplementary Figure S1.

Supplementary Figure S2.

Supplementary Legends.

Supplementary Video S1.

Supplementary Video S2.

Supplementary Video S3.
