# Supplementary figures and images for: Role of senescent cells in the motile behavior of active, non-senescent cells in confluent populations
Source: Sci Rep. 2022 Mar 9;12:3857. doi: 10.1038/s41598-022-07865-2 (PMC8907270; doi:10.1038/s41598-022-07865-2)

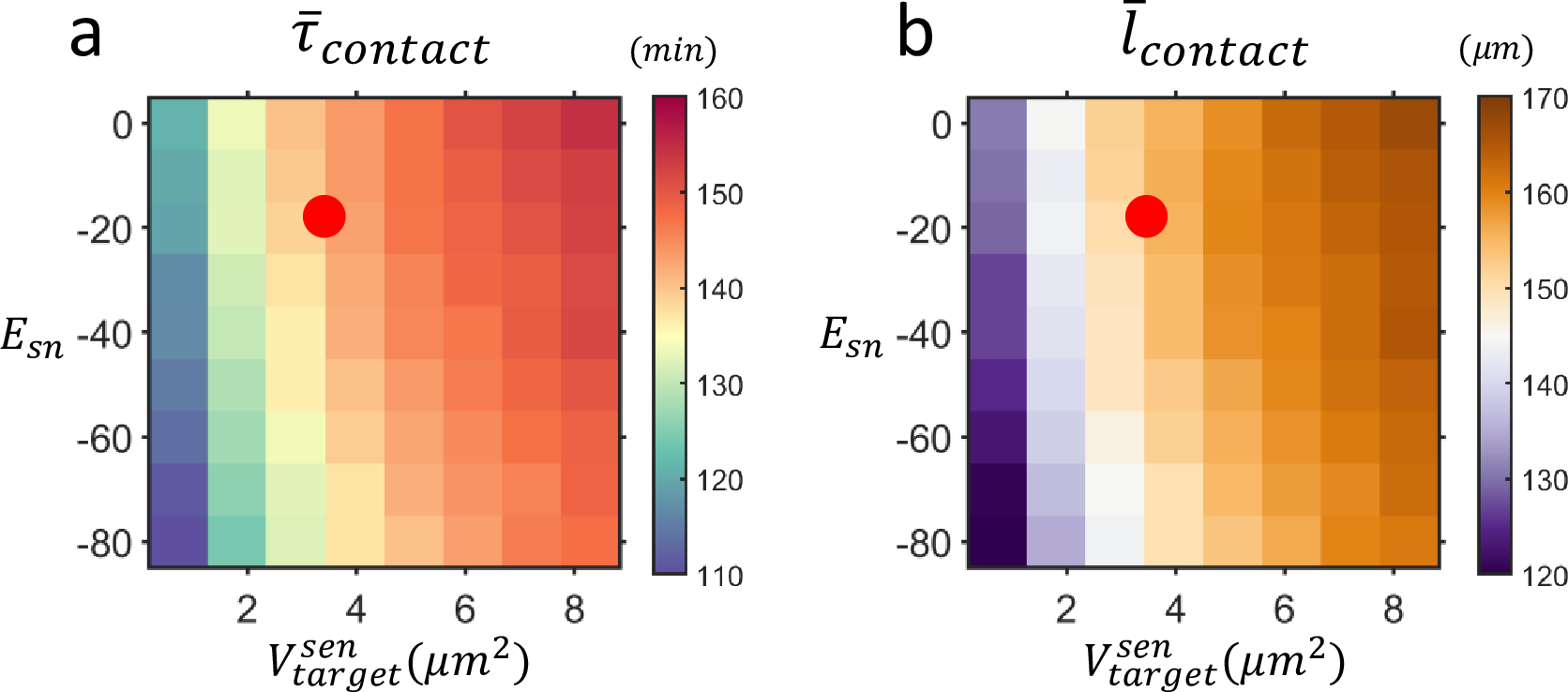

Supplement: Supplementary file 1 — Supplementary Figure S1. [file 41598_2022_7865_MOESM1_ESM.tif]

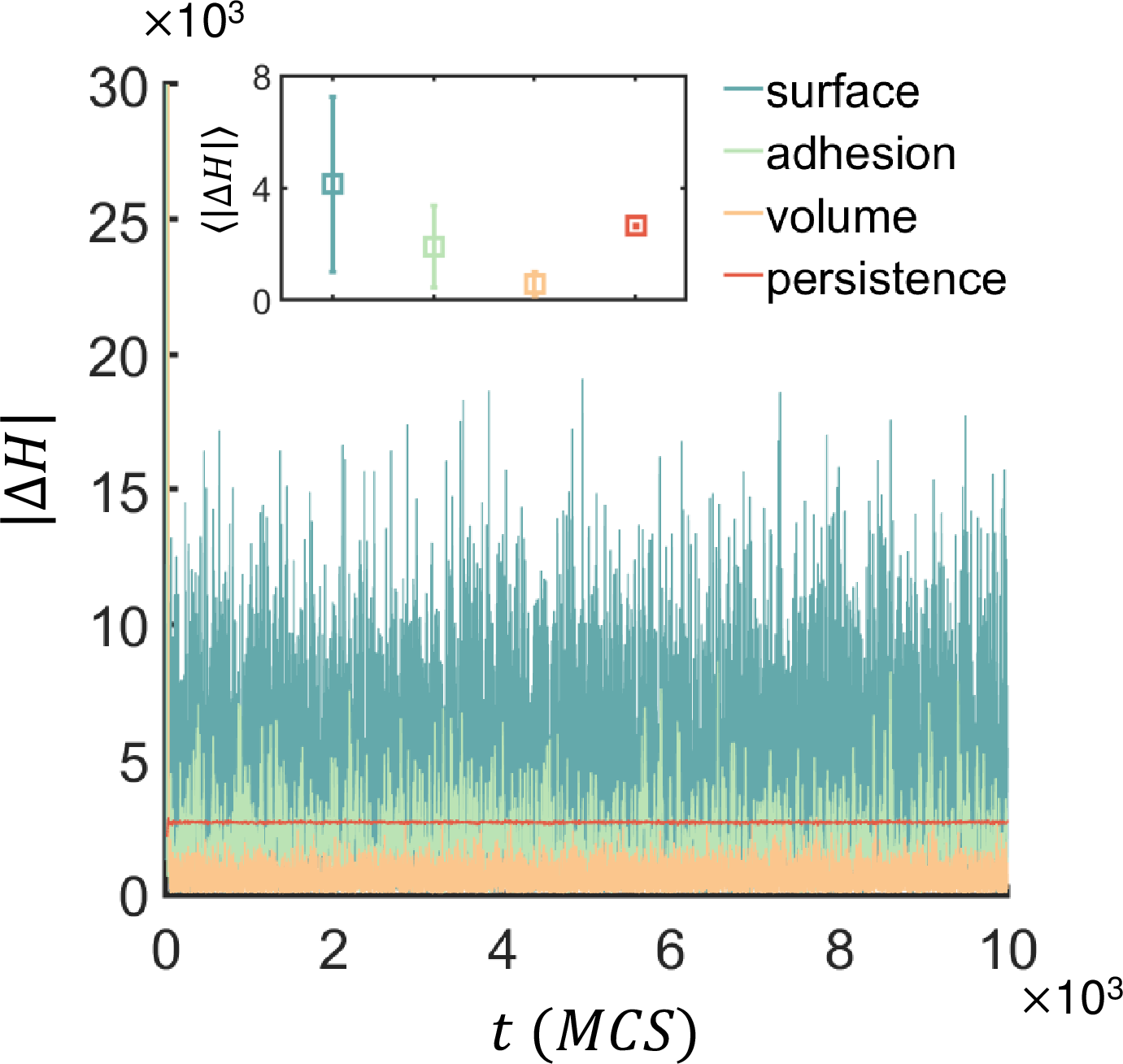

Supplement: Supplementary file 2 — Supplementary Figure S2. [file 41598_2022_7865_MOESM2_ESM.tif]
