# Supplementary material for: Role of senescent cells in the motile behavior of active, non-senescent cells in confluent populations
Source: Sci Rep. 2022 Mar 9;12:3857. doi: 10.1038/s41598-022-07865-2 (PMC8907270; doi:10.1038/s41598-022-07865-2)
Supplement: Supplementary file 3 — Supplementary Legends. [file 41598_2022_7865_MOESM3_ESM.docx]

Supplementary material

Role of senescent cells in the motile behavior of active, non-senescent cells in confluent populations

Thamara Liz Gabuardi^1,2^, Hyun Gyu Lee^1,2^, and Kyoung J. Lee^1^*

^1^Department of Physics, Korea University, Seoul, Korea

*Corresponding author

Email: kyoung@korea.ac.kr (KJL)

**Fig. S1. Heat map of population mean of (a)** $\boldsymbol{\tau}_{\boldsymbol{contact}}$ **and (b)** $\boldsymbol{l}_{\boldsymbol{contact}}$**.**  Note that both $\tau_{contact}$ and $l_{contact}$ increase monotonically as a function of $E_{sn}$ (and $V_{target}^{sen}$). The red dots mark the location of experimentally relevant values of $E_{sn}$ and $V_{target}^{sen}$.

**Fig. S2. Estimation of the amplitude of energy changes over one MC time step.** For the experimentally relevant CPM simulation, the role of surface energy change is the most significant of all four different energy types.

**Supplementary Video 1:** A normal MDA-MB-231 cell encircling a hugely expanded senescent cell in a 2D cell culture.

**Supplementary Video 2:** A cellular Potts model simulation of an active cell moving along a non-actively moving (senescent cell-like) cell.

**Supplementary Video 3:** A cellular Potts model simulation of actively moving cells about a huge senescent cell.
